# Highly Crystalline and Oriented Thin Films of Fully Conjugated 3D‐Covalent Organic Frameworks

**DOI:** 10.1002/anie.202505799

**Published:** 2025-07-10

**Authors:** Ignacio Munoz‐Alonso, Derya Bessinger, Stephan Reuter, Marcello Righetto, Laura Fuchs, Markus Döblinger, Dana D. Medina, Frank Ortmann, Laura M. Herz, Thomas Bein

**Affiliations:** ^1^ Department of Chemistry and Center for Nanoscience (CeNS) University of Munich (LMU) Butenandtstraße 11 (E) 81377 Munich Germany; ^2^ Department of Physics Clarendon Laboratory University of Oxford Parks Road Oxford OX1 3PU UK; ^3^ Department of Chemistry TUM School of Natural Sciences and Atomistic Modeling Center Munich Data Science Institute Technical University of Munich (TUM) 85748 Garching b. München Germany

**Keywords:** 3D‐fully conjugated COFs, Electron mobility, GIWAXS, Photoconductivity, Thin films

## Abstract

Fully conjugated 3D covalent organic frameworks (COFs) are a newly emerged class of materials that expands reticular chemistry to extended electron delocalization for optoelectronic applications. To overcome the limitations of sp^3^‐connected 3D frameworks, the pseudo‐tetrahedral motif cyclooctatetrathiophene (COTh) has gained attention for forming fully conjugated 3D COFs. We report on a novel COTh building block, featuring functional formyl groups directly attached to the core's conjugated thiophenes. The modulation synthesis approach with mono‐functionalized inhibitors enables the formation of COTh‐1P COF, which exhibited remarkable crystallinity and permanent porosity. By following this approach and by optimizing the synthesis conditions for the solvothermal growth of thin films, we fabricated the first preferentially oriented conjugated 3D COF films on various substrates without pre‐functionalization. With these thin films, optical pump terahertz probe studies allowed us, for the first time with 3D‐fully conjugated COFs, to provide insights into the excited state and charge‐carrier dynamics of these unique organic frameworks. Low effective masses are discovered for valence and conduction bands by density functional theory simulations. The ability to create crystalline and oriented films of fully π‐conjugated 3D COTh‐based COFs on non‐modified substrates is expected to open the way for integration of such frameworks into diverse optoelectronic applications.

## Graphical abstract

This work introduces a fully conjugated, 3D covalent organic framework (COF) based on cyclooctatetrathiophene (COTh); the synthesis employs modulated conditions to achieve thin films with high crystallinity. The COTh‐1P COF shows potential for optoelectronic applications, thanks to its π‐conjugated structure, the preferential film orientation, and charge dynamics insights obtained from optical pump terahertz probe studies.

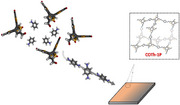

## Introduction

Employing the principles of reticular chemistry, the linkage of rigid organic building blocks through condensation reactions gives rise to a distinct category of materials featuring crystalline open porous frameworks. These resulting covalent organic frameworks (COFs) can manifest as either 2D^[^ ^1^, ^2^, ^3^ ^]^ or 3D structures,^[^ ^4^, ^5^ ^]^ depending on the topology and architecture of the precursors chosen. Since the pioneering discovery of 3D COFs in 2007 by El‐Kaderi and colleagues,^[^ ^6^ ^]^ 3D COFs have attracted growing interest owing to their intriguing properties and broad and versatile potential for several applications. These porous and chemically stable frameworks have found utility in diverse areas, such as gas storage and separation,^[^ ^7^, ^8^, ^9^, ^10^ ^]^ dye removal,^[^ ^11^, ^12^ ^]^ ion exchange,^[^ ^12^, ^13^ ^]^ drug delivery,^[^ ^14^ ^]^ and catalysis.^[^ ^15^, ^16^ ^]^

The majority of building blocks employed in the creation of 3D COFs typically consist of (pseudo)tetrahedral nodes like tetraphenylmethane,^[^ ^6^, ^17^ ^]^ tetraphenylsilane,^[^ ^7^, ^18^ ^]^ or adamantane.^[^ ^15^, ^19^ ^]^ These nodes are linked to multidentate counterparts with various geometries, resulting in frameworks of different topologies.^[^ ^20^ ^]^ However, the presence of sp^3^‐hybridized carbon atoms in these nodes disrupts continuous π‐conjugation, which is crucial for efficient charge transport and desirable optical properties in materials like organic semiconductors.

To overcome these challenges and establish a fully conjugated 3D network, the pseudo‐tetrahedral node cyclooctatetrathiophene (COTh), featuring an sp^2^ carbon‐conjugated structure, has recently been incorporated into COFs.^[^ ^21^, ^22^, ^23^, ^24^, ^25^ ^]^ Initially reported by Kauffmann et al. in 1978,^[^ ^26^ ^]^ COTh, with its saddle‐shaped configuration, has remained a focal point in subsequent research.^[^ ^27^, ^28^, ^29^, ^30^ ^]^ COTh features a central [8]annulene moiety as a nonplanar 8π‐conjugated skeleton formed by four thiophenes connected at the 2‐ and 3‐positions. Single‐crystal X‐ray analysis has confirmed the “saddle” form of COTh,^[^ ^31^, ^32^, ^33^ ^]^ making this molecule a promising new building unit for the development of fully conjugated COFs. Although COTh‐based building blocks were successfully integrated into the highly ordered structures of COFs and proved their extended π‐conjugation with COF materials exhibiting moderate conductivity and electron mobility values,^[^ ^21^, ^22^, ^23^, ^24^ ^]^ the development of preferentially oriented thin films has not been reported yet. However, transferring the features of fully conjugated COTh‐COFs from hitherto randomly oriented bulk powder samples to the ordered and well‐defined morphology of grown thin films is expected to further accentuate the capabilities of the COTh building blocks and additionally expand the field of possible applications.

Oriented thin films of COFs already show great potential as the active material in various applications for 2D COFs, such as photovoltaics,^[^ ^34^, ^35^, ^36^ ^]^ water splitting,^[^ ^37^, ^38^, ^39^ ^]^ photodetection,^[^ ^40^ ^]^ acidochromism,^[^ ^41^ ^]^ solvatochromism,^[^ ^42^ ^]^ and electrochromism.^[^ ^43^ ^]^ However, preferentially oriented thin films of 3D COFs have not been reported to date. Furthermore, the few existing 3D COF films on substrates are mainly fabricated with pre‐modified substrate surfaces containing functional groups on which the COFs specifically grow.^[^ ^44^, ^45^, ^46^ ^]^ Since many electrochemical and electronic applications require direct contact of the COFs with a conductive substrate, oriented growth of ordered structures on non‐functionalized substrates would be highly desirable.

The crystallinity of COFs plays a key role in determining their quality and functionality. Hence, it is crucial to tailor reaction conditions to facilitate controlled and high‐quality crystal growth. For example, the crystallinity of the active COF materials has been shown to play a crucial role in applications such as gas separation and storage,^[^ ^8^, ^47^, ^48^ ^]^ catalysis,^[^ ^49^, ^50^ ^]^ filtration,^[^ ^51^ ^]^ sensors,^[^ ^41^, ^52^, ^53^ ^]^ electrochromism,^[^ ^43^ ^]^ and optoelectronics,^[^ ^34^, ^35^, ^52^ ^]^ which require a meticulously ordered and open porous structure.

To exert better control over morphology and to enhance the crystallinity of COFs, control of kinetic barriers^[^ ^54^ ^]^ or the use of modulating agents has been demonstrated to be key.^[^ ^5^ ^]^ In the latter approaches, monofunctionalized modulators act as end caps during COF growth, dynamically competing with multifunctional constituents of the framework. Consequently, they alter the crystal growth rate. Coupling these processes with the intrinsic defect correction processes given by the reversible bond formation promotes the controlled growth of well‐ordered and highly crystalline domains and even single crystals. This modulation approach has proven successful in the syntheses of both 2D and 3D COFs, demonstrating the positive impact on their crystallinity.^[^ ^5^, ^55^, ^56^, ^57^ ^]^

Alternatively, controlled nucleation has been employed in a seeded growth approach to produce single‐crystalline 2D COFs.^[^ ^58^ ^]^ Here, the gradual addition of monomers to preformed nanoparticles in a second polymerization step increased the single crystal domain size up to 1.5 µm, demonstrating the positive impact of controlling early nucleation dynamics in COF growth.

In this work, we developed a novel 3D‐fully conjugated COF with an incorporated sp^2^ carbon‐conjugated COTh building block serving as a pseudo‐tetrahedral node. Besides the already known extended building blocks with bridging phenylene moieties attached to the core, here we designed a more compact version of the COTh building block with the functional groups required for the COF imine bond formation, namely formyl groups, directly fused to the four thiophenes of the [8]annulene core, thereby enabling the formation of compact 3D frameworks with reduced flexibility and higher π‐electron density. We then transferred the modulation approach to the in situ 3D COF thin film fabrication to slow down the nucleation rate, impede the fast precipitation of bulk material, and thereby promote the controlled growth of crystalline COF on the substrate surface. This led to the first preferentially oriented 3D COF films on various substrates, which eventually showed promising electrical characteristics.

## Results and Discussion

For the construction of the sp^2^ carbon‐conjugated 3D COF, a new pseudo‐tetrahedral, saddle‐shaped COTh building block (COTh(CHO)_4_) with terminal aldehyde groups was synthesized. These terminal aldehydes enable the reversible imine bond formation during the COF synthesis. The saddle‐shaped building block was synthesized starting from tetrabrominated bithiophenes that were protected at the α‐positions and fused at the vacant bromine positions via cyclodimerization. The synthesis of the COTh(CHO)_4_ was conducted following a four‐step protocol (Figure S2), starting with selective formylation of 3,3′,5,5′‐tetrabromo‐2,2′‐bithiophene in the presence of *n*‐BuLi, yielding compound **1**.^[^ ^59^ ^]^ Acetal protection of the aldehyde groups via neopentyl glycol produced compound **2** and allowed for the formation of the [8]annulene core **3** via copper‐catalyzed Ullman cross‐coupling, followed by deprotection with trifluoroacetic acid (TFA), thus affording the novel tetra‐aldehyde building block COTh(CHO)_4_ in overall good yields. The successful synthesis and purity of the building block were confirmed by ^1^H‐NMR, ^13^C‐NMR, and mass spectromety (MS) (see the Supporting Information, Section 2).

The presence of four aldehydes attached to the flexible, saddle‐shaped central core enables condensation with primary amines, forming imine bonds between building blocks and allowing for the combination of the tetrahedral node with a variety of complementary counterparts. Here, the length of the linear diamines serving as linkers was deliberately kept short to increase π‐electron density and to reduce framework flexibility with possible stability issues.

**Figure 1:**
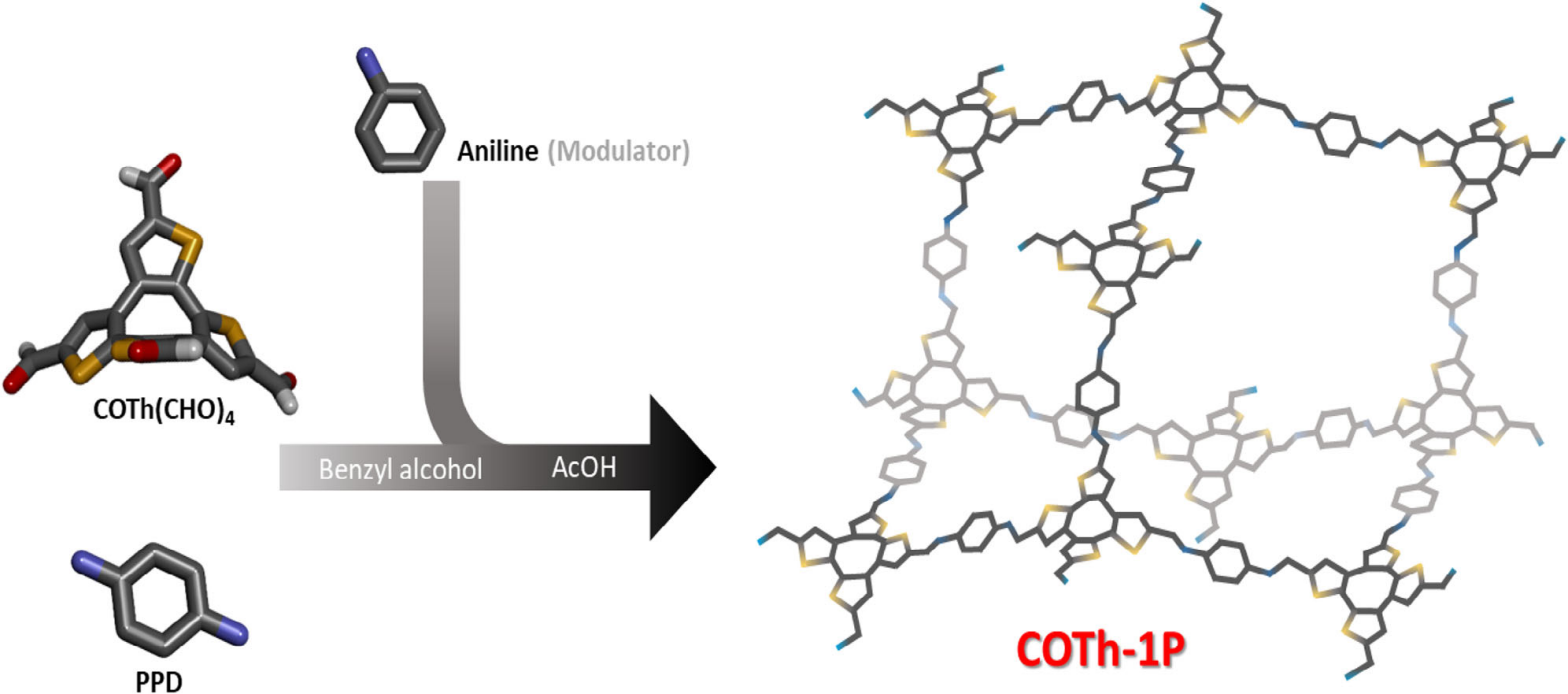
Synthesis of the new COTh‐1P COF. The co‐condensation of the tetrahedral building block COTh(CHO)_4_ with *p*‐phenylene diamine (PPD) leads to the 3D COF named COTh‐1P. To enhance the crystallinity, the COF growth was controlled by adding the monofunctionalized modulator aniline to the synthesis. The resulting framework crystallized in the diamond topology and is constructed of interpenetrated adamantane‐like cages (color scheme: C: dark gray; S: yellow; N: blue).

The COF COTh‐1P was synthesized under acid‐catalyzed solvothermal conditions and obtained as a red powder by co‐condensation of the COTh(CHO)_4_ and *p*‐phenylenediamine (PPD) building blocks at a 1:2 molar ratio (see Figure 1). To improve the crystallinity of the 3D framework material, we introduced mono‐functionalized benzene molecules, such as aniline, during the COF synthesis, serving as a modulator (Supporting Information, Section 3.3). Optimal results were achieved through a process involving partial dissolution of the modulator and its corresponding aldehyde‐containing counterpart in benzyl alcohol solvent, followed by the addition of acetic acid at room temperature. Subsequently, the second building block was introduced, and the reaction mixture was subjected to solvothermal conditions at 120 °C in a sealed Pyrex reaction tube.

**Figure 2:**
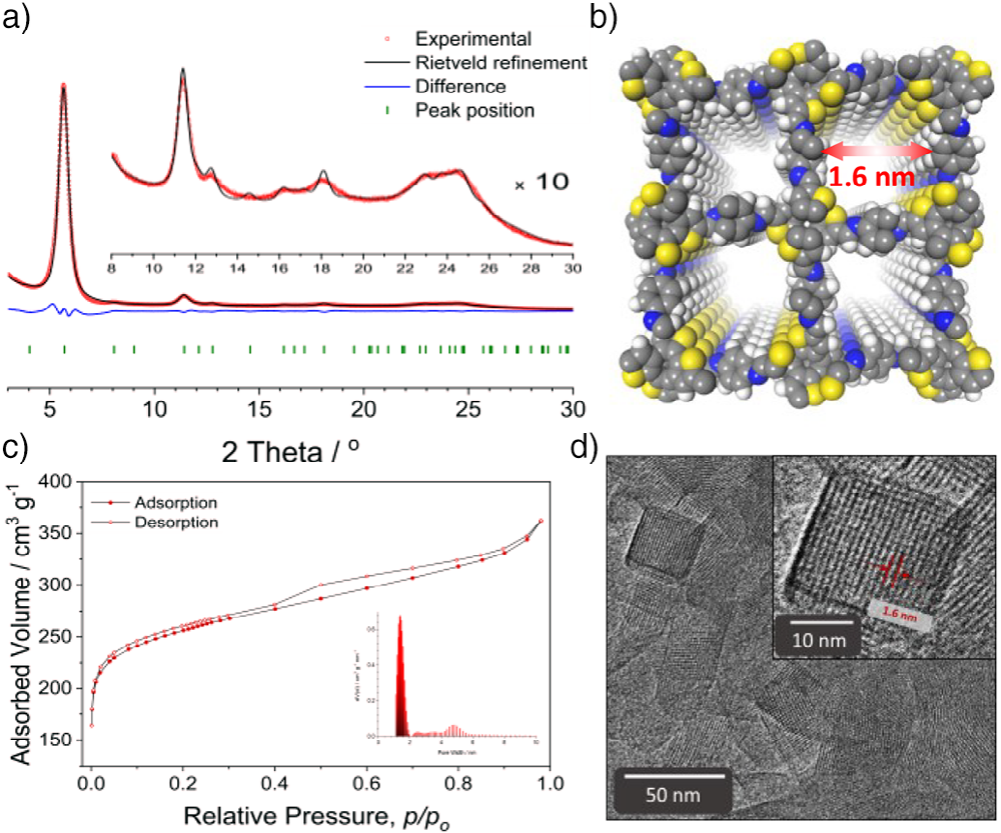
Structural characterization of the COTh‐1P COF: a) Experimental PXRD pattern of the COF bulk material (red dots) and Rietveld refinement (black line) using a structure model in the tetragonal *I*4_1_ space group. The peak positions are indicated by cyan ticks, and the difference plot (blue line) shows an overall good fit between the experimental and refined data (R_wp_ = 6.17%. Inset: tenfold magnification of the 8° < 2*θ* < 30° region. b) Refined COF structure simulation with a diamond topology showing square‐shaped pores when viewed along the *c*‐axis and exhibiting a sevenfold interpenetration (dia‐c7). c) Type Ib nitrogen sorption isotherm recorded at 77 K with the NLDFT‐based pore size distribution modeled for cylindrical pores, showing a maximum at 1.1 nm, which corresponds very well to the simulated average pore size of 1.2 nm. d) High‐resolution TEM image of the polycrystalline powder. Inset: A well‐ordered single crystallite oriented with view along the pore channels in c‐direction shows the square‐shaped pores and a periodicity of 1.6 nm in excellent agreement with the simulated periodicity of the refined structure (red arrows).

The resulting COF based on COTh, synthesized using this modulation approach, exhibited high crystallinity as confirmed by powder X‐ray diffraction (PXRD), which revealed several well‐defined reflections, particularly at lower diffraction angles (see Figure 2a). The distinct reflections observed in the higher‐order range of the X‐ray diffractograms, along with the absence of a visible amorphous background, indicate a highly ordered periodic structure. Previous studies employing aniline as a modulator in the synthesis of imine‐linked 3D COFs have demonstrated significant improvements in crystallization, yielding single crystals of up to 60 µm.^[^ ^5^ ^]^ In this context, the COF nucleation and growth are slowed down by the presence of monofunctional reaction competitors that act as modulators, interrupting rapid imine formation from the multi‐functional building units and therefore enhancing crystallinity. The similar reactivities between the COF building units and aniline optimize the inhibitor's effect on the crystallization processes. Hence, an excess of terminating amine monomer during the reaction increases the reversibility of imine‐bond formation, aiding in defect correction during framework construction and promoting COF crystal growth.^[^ ^54^ ^]^

The formation of a highly ordered structure of the novel 3D COTh‐1P COF core unit was first confirmed by PXRD analysis (Figure 2a), which provided intense reflections at low (5° < 2θ < 11°) and high diffraction angles (> 15°). According to the chemical components and their predictable spatial arrangement, a structure model in the tetragonal *I*41 space group was constructed. The structure model can be described by a diamond topology (dia‐c7) with a sevenfold interpenetration. In a view along the *c*‐axis, the interpenetrated diamond net shows square‐shaped 1D pore channels with a simulated periodicity of 1.6 nm (Figures 2b and S15).

The simulated diffraction pattern of the pristine model shows a good agreement with the experimental one. Prominent reflections are centered at 2*θ* = 5.7°, 11.42°, and 18.1°, corresponding to hkl (020), (040), and (260) planes, respectively. The structure model was successfully refined by the Rietveld method,^[^ ^60^ ^]^ showing a good approximation to the intensity distribution of the experimental PXRD pattern (R_wp_ = 6.17%) and indicating that the structure model is essentially correct. The refined lattice constants are *a* = *b* = 30.96 Å, *c* = 4.59 Å, and *α* = *β* = *γ* = 90°, the refined domain sizes perpendicular and along the fourfold axis are 14 and 6 nm, respectively.

**Figure 3:**
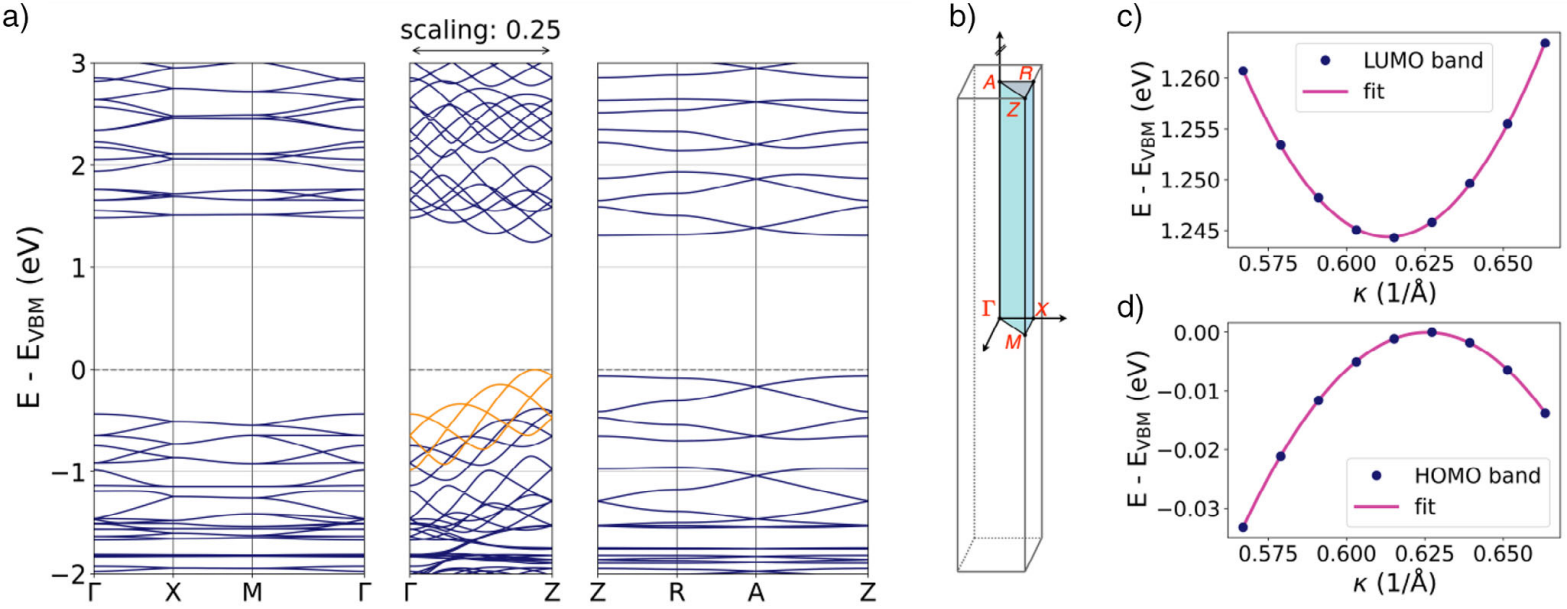
a) Band structure of COTh‐1P COF showing significant out‐of‐plane dispersion along the Γ–Z direction and a sevenfold oscillation in energy in the valence and conduction bands, consistent with the framework's interpenetrated structure. The band gap energy is estimated to be 1.25 eV between the apexes (*cf*. Supplementary materials for details). The valence band maximum (VBM) is selected as energy zero. b) Brillouin zone of the primitive tetragonal unit cell with high‐symmetry points. c) and d): Close‐up of band‐edge regions, showing indirect band character close to the Z‐point.

Density functional theory (DFT) calculations, including van der Waals corrections, were performed to relax the COTh‐1P structure (see Supporting Information for details). Several initial geometries were tested, and the final equilibrium structure was selected based on comparing their total energies. We next studied the band structure of COTh‐1P (Figure 3a), which reveals moderate in‐plane band dispersions, indicative of limited charge carrier mobility within the layers. In contrast, along the out‐of‐plane direction (Γ–Z), both valence and conduction bands exhibit substantial bandwidths reaching or even exceeding 1 eV, suggesting the possibility of enhanced electronic transport across layers. The band dispersion gives rise to an indirect bandgap, estimated to be 1.25 eV. The indirect character is, however, due to only a slight offset in momentum between the valence and conduction band extrema, occurring away from the Γ point (Figure 3c,d, respectively). The most interesting feature of the band structure is the emergence of multiple sets of bands with distinct sevenfold oscillation patterns (highlighted exemplarily for the valence band edge in orange), which correlates with the sevenfold lattice interpenetration observed in the COF structure. The topmost valence bands are localized on the COTh cores, and their strong dispersion indicates promising electronic transport properties perpendicular to the molecular planes, enabled by the COTh‐1P COF interpenetrated architecture. This is further corroborated by low band‐effective masses of 0.5 *m_e_* and −0.4 *m_e_* (*m_e_* the electron mass) along the stacking direction for electrons and holes, respectively.

Nitrogen sorption measurements conducted at 77 K (Figure 2c) confirmed the accessibility of the nanoporous COTh‐1P, revealing a type I(b) isotherm with a steep uptake below *p/p*_0_ = 0.05 and a Brunauer–Emmett–Teller (BET) surface area of 955 m^2^ g^−1^ with a total pore volume of 0.53 cm^3^ g^−1^. The pore size distribution was determined using a nonlocal density functional theory (NLDFT) model for cylindrical pores and showed a peak pore diameter of 1.1 nm, close to the simulated average pore dimension of 1.2 nm. High‐resolution transmission electron microscopy (HR‐TEM) images of the polycrystalline COF bulk material show the formation of highly textured crystallites in the size range of around 25 nm (Figure 2d). Evaluation of a single COF domain with its 1D rectangular pores oriented along the viewing direction reveals a periodicity of 1.6 nm, which is in excellent agreement with the periodicity of the simulated structure model.

Employing the in situ film growth combined with controlled nucleation during the solvothermal synthesis by means of the modulation approach allowed for the fabrication of homogenous and preferentially oriented 3D COTh‐1P COF thin films on various substrates. Here, we note that the modulator plays a pivotal role in decreasing the crystallization rate, thus slowing down the COF formation, thereby reducing the amount of material that would otherwise precipitate quickly and could thus not be deposited as crystalline COF material on the substrate surface. The mono‐functionalized modulator was added to the synthesis batch prior to the insertion of the substrate with the to‐be‐coated surface face‐down in a polytetrafluoroethylene (PTFE) holder (see the Supporting Information, Section 3.2 for experimental details, Table S3 and Figure S9).

**Figure 4:**
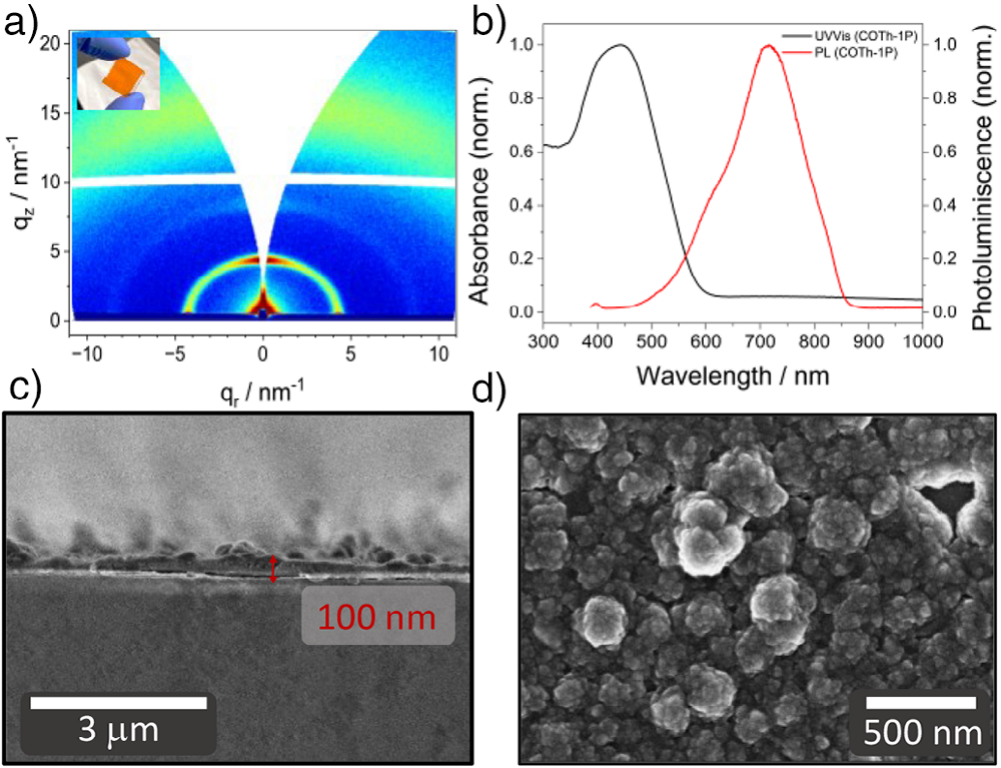
Characterization of COTh‐1P thin films. a) GIWAXS pattern of a COTh‐1P film grown on fused silica with high intensities at low *q_r_*‐ and *q_z_*‐values, respectively, indicating a preferentially oriented thin film. Inset: Photographic picture of the COTh‐1P thin film on a fused silica substrate. b) UV–vis absorption spectra (black) and photoluminescence (red) of the COTh‐1P COF as a film. The distinctly redshifted absorption of the COF, compared to the COTh(CHO)_4_ linker reported in the Supporting Information, indicates the successful incorporation of the building blocks into the imine‐linked structure, leading to increased electronic conjugation. c) Cross‐sectional SEM image of the COTh‐1P film on fused silica substrate with a thickness of around 100 nm. d) Top‐view SEM image of the COTh‐1P film on fused silica confirming the preferential orientation of the material on the substrate.

In contrast to previously reported 3D COF thin films,^[^ ^61^, ^62^ ^]^ the substrates do not require modification of the surface for a homogeneously grown COF layer with controllable and constant thickness. This establishes new possibilities for fabricating 3D COF thin films with direct contact on specific substrates required for different applications. COTh‐1P COF thin films were grown on three substrates, i.e., fused silica, indium tin oxide (ITO), and partially coated ITO, that were then subjected to optical and electrochemical measurements. After the solvothermal synthesis, all substrates are covered with homogeneously grown, crystalline COF thin films with a preferential orientation that is confirmed by grazing‐incidence wide‐angle X‐ray scattering (GIWAXS, Figure 4a). With intensity maxima close to the sample horizon at *q_z_* ≥ 0 nm^−1^ and perpendicular to it at *q_r_* ≥ 0 nm^−1^, the GIWAXS pattern indicates a crystalline film with two main orientations along the tetragonal crystal axes. Top‐view and cross‐sectional scanning electron microscopy (SEM) images confirm the homogeneous growth of a 100 nm thin film with high COF density on top of the substrate (Figures 4c,d and S13).

Cyclic voltammetry (CV) measurements of the COTh‐1P COF thin film were conducted with a three‐electrode setup. To this end, the COF was grown on a conductive ITO substrate that was then applied as the working electrode. A platinum and a silver wire functioned as the counter electrode and the quasi‐reference electrode, respectively, and 0.1 M tetrabutylammonium hexafluorophosphate (TBAPF_6_) in acetonitrile was used as the electrolyte. The CV curves were recorded at a scan rate of 50 mV s^−1^ and calibrated against the ferrocene fc/fc^+^ redox couple. The measurements show a well‐defined oxidation wave at 0.11 V versus fc/fc^+^ with only minimal drift over four cycles, confirming the reversibility of the redox processes and the stability of the COF material in this potential range (Figure S12).

Optical characterization via UV–vis and photoluminescence (PL) measurements reveals a broad optical absorption band of the COTh‐1P COF with a maximum at 445 nm and a Stokes‐shifted PL peaking at 716 nm (Figure 4b). The COF's optical band gap was estimated to be 2.32 eV from the absorption onset via Tauc plot for a direct transition (Figure S11). A larger optical band gap compared to the electronic band gap suggests that the optical spectra are not governed by the frontier electronic states—a behavior typically observed in organic systems with charge‐transfer excitation character.^[^ ^63^ ^]^

These findings demonstrate the great potential of the modulation approach for growing high‐quality oriented 3D COF thin films in a controlled manner on specific substrates to minimize structural irregularities that can strongly impact the transport properties of COFs.^[^ ^64^ ^]^

**Figure 5:**
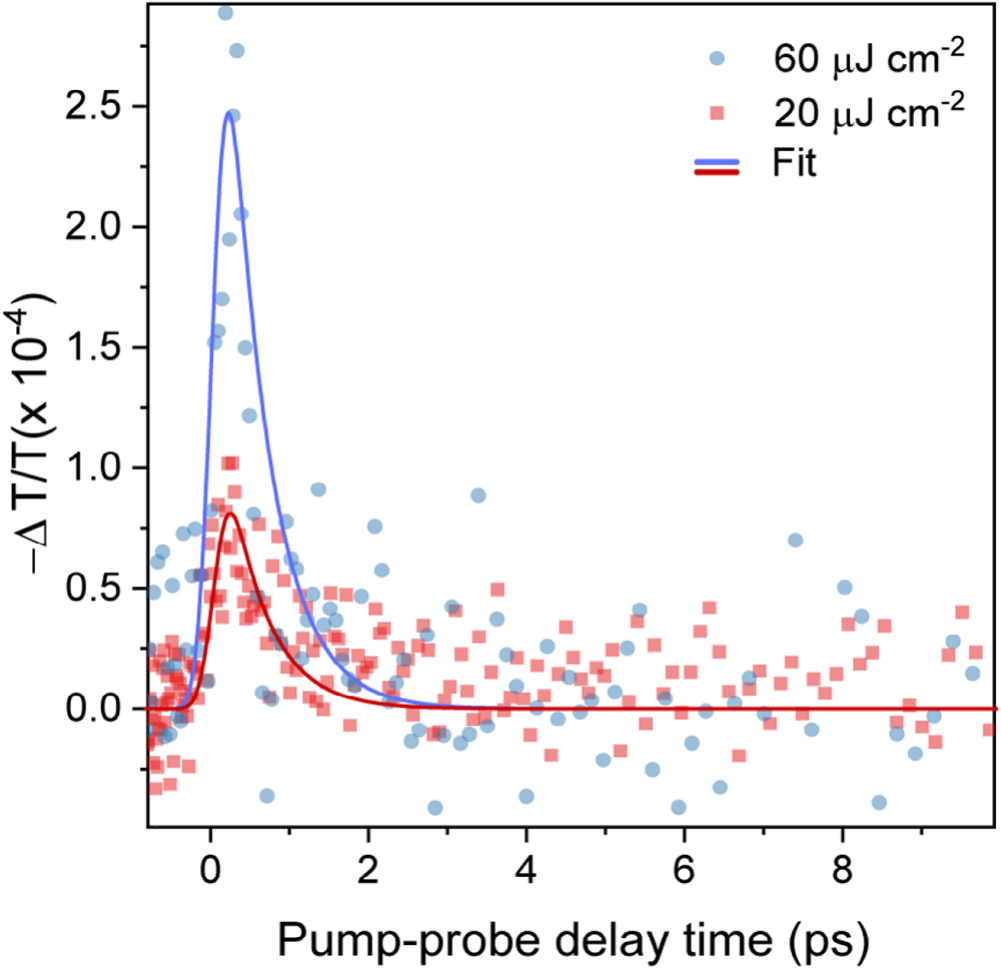
Fractional change in the transmitted THz‐field amplitude for COTh‐1P COF films, proportional to the THz photoconductivity, plotted as a function of time after excitation with a 400 nm pulse at a fluence of 60 µJ cm^−2^ (blue circles) and 20 µJ cm^−2^ (red squares). Solid lines represent fits to a single exponential decay model described in detail in the Supporting Information.

To gain further insights into the nature and dynamics of photoexcited charge carriers, we studied the time‐resolved photoconductivity of COTh‐1P‐COF by using optical‐pump THz‐probe (OPTP) spectroscopy. In our OPTP experiments, charge carriers are optically generated in a COF thin film by an ultrafast 400 nm pump pulse, and the resulting photoconductivity is probed by a single‐cycle THz pulse polarized in the substrate plane. Figure 5 shows the fluence‐dependent OPTP signal (expressed as the fractional change in the transmitted THz‐field amplitude, ‐*ΔT/T*, proportional to the photoconductivity) measured for a COTh‐1P‐COF film.

We attribute the ultrafast rise of the OPTP signal to the transient generation of free electron and hole pairs upon photoexcitation. The observed photoconductivity signal is proportional to the photogenerated charge‐carrier density *n* and depends on two factors: the photon‐to‐charge branching ratio *ϕ* (i.e., the fraction of free carrier pairs generated per absorbed photon, varying between 0 and 1) and the electron‐hole sum mobility *μ*. Therefore, from OPTP measurements, we can extract the lower bound of the electron‐hole sum mobility corresponding to the branching ratio‐mobility product *ϕμ*, also referred to as effective mobility.^[^ ^65^ ^]^ The average effective mobility value extracted for COTh‐1P‐COF films is *ϕμ* = 0.18 ± 0.03 cm^2^ V^−1^ s^−1^. Furthermore, we observe an ultrafast photoconductivity decay with a decay rate *k*_d _≈ 2 ps^−1^, similar to what was previously reported for different COFs.^[^ ^66^ ^]^ In addition, the frequency‐dependence of the photoconductivity (Figure S1) shows deviations from the Drude conductivity model.^[^ ^67^ ^]^ The observed frequency dependence, i.e., positive real and negative imaginary components of the photoconductivity with increasing frequency, is characteristic of dispersive transport in a disordered medium.^[^ ^68^ ^]^ Following the approach developed by Milot et al.,^[^ ^69^ ^]^ we assessed these deviations via a “Drude factor” and obtained a value of ∼0.7, significantly different from the perfect Drude behavior indicated by a Drude factor value of 1 (see Supporting Information).

Our results can be rationalized by considering the nature of photogenerated carriers in organic conjugated materials. Although sub‐ps decays previously observed in different COFs have also been attributed to trapping and localization,^[^ ^66^ ^]^ we note that similar OPTP dynamics have been widely reported for other excitonic materials, e.g., conjugated polymers.^[^ ^70^, ^71^ ^]^ Due to high effective masses, low dielectric constants, and strong coupling with vibrations, the primary photoexcited species in COFs and other organic semiconductor materials are coulombically bound electron‐hole pairs (i.e., excitons). Therefore, we expect highly energetic pump pulses to generate quasi‐free electron and hole pairs, which will dissipate the excess energy and form excitons, yielding the observed ultrafast rise and sub‐ps decay of the photoconductivity.

Furthermore, the excitonic nature of these COFs also suggests that the photon‐to‐charge branching ratio is potentially small, as previously demonstrated for other organic semiconductors.^[^ ^68^, ^71^ ^]^ We note here that mobilities in COTh‐1P‐COF could, therefore, be considerably higher than the effective (i.e., weighted by the branching ratio) value extracted here, which represents a lower boundary for charge‐carrier mobility. We stress that a dramatic variation in the reported mobilities could result from different approaches in the analysis (e.g., via fitting of the frequency‐dependent complex photoconductivity versus estimating the sheet photoconductivity) of optical‐pump THz probe data.

## Conclusion

We have developed highly crystalline 3D COFs employing the pseudo‐tetrahedral and sp^2^ carbon‐conjugated cyclooctatetrathiophene building block in modulator‐controlled condensation reactions. The novel compact variant of the COTh building block functionalized with aldehydes directly connected to the core was covalently linked to linear phenylene moieties to form the COTh‐1P COF with exceptional crystallinity. The interpenetrated framework with diamond topology exhibits accessible square‐shaped channels, which was also confirmed by nitrogen sorption analysis and transmission electron microscopy.

The synthetic modulation approach enabled the formation of homogenous 3D COTh‐1P COF thin films on various substrates that do not require a prior modification of their surface. The growth of a thin film with a preferential orientation and controllable thickness allows for the direct transfer of the intrinsic properties of COTh‐1P to specific application fields and therefore increases their versatility. Present work in our laboratories aims to extend and tune the optoelectronic features of 3D conjugated COFs by means of building block design and linkage chemistry.

## Supporting Information

The authors have cited additional references within the Supporting Information.^[^ ^72^, ^73^, ^74^, ^75^, ^76^, ^77^, ^78^, ^79^, ^80^, ^81^, ^82^, ^83^, ^84^, ^85^, ^86^, ^87^ ^]^

## Conflict of Interests

The authors declare no conflict of interest.

## Supporting information



Supporting Information

## Data Availability

The data that support the findings of this study are available in the Supporting Information of this article.
