# Supplementary material for: Highly Crystalline and Oriented Thin Films of Fully Conjugated 3D‐Covalent Organic Frameworks
Source: Angew Chem Int Ed Engl. 2025 Jul 10;64(34):e202505799. doi: 10.1002/anie.202505799 (PMC12363643; doi:10.1002/anie.202505799)
Supplement: Supplementary file 1 — Supporting Information [file ANIE-64-e202505799-s001.docx]

**Highly Crystalline and Oriented Thin Films of Fully Conjugated 3D-Covalent Organic Frameworks**

Ignacio Munoz-Alonso,^1, ‡^ Derya Bessinger,^1, ‡^ Stephan Reuter,^1^ Marcello Righetto,^2^ Laura Fuchs,^3^ Markus Döblinger,^1^ Dana D. Medina,^1^ Frank Ortmann,^3^ Laura M. Herz,^2^ and Thomas Bein^1*^

*^‡^These authors contributed equally to this work.*

^1^Department of Chemistry and Center for NanoScience (CeNS), University of Munich (LMU), Butenandtstraße 5-13, 81377 Munich, Germany
^2^ Clarendon Laboratory, Department of Physics, University of Oxford, Parks Road, OX1 3PU, Oxford, United Kingdom.

^3^ Department of Chemistry, TUM School of Natural Sciences and Atomistic Modeling Center, Munich Data Science Institute, Technical University of Munich, 85748 Garching b. München (Germany)

Table of contents

[1 Methods 2](#_Toc202771152)

[2 Linker synthesis 6](#_Toc202771153)

[3 COF Synthesis 11](#_Toc202771154)

[3.1 COTh-1P COF powder 11](#_Toc202771155)

[3.2 COTh-1P film 11](#_Toc202771156)

[3.3 Effect of modulator 12](#_Toc202771157)

[4 Optical Characterization 16](#_Toc202771158)

[5 Electrochemical Measurements 17](#_Toc202771159)

[6 Scanning electron microscopy 17](#_Toc202771160)

[7 Fourier-transform infrared spectroscopy (FT-IR) 18](#_Toc202771161)

[8 Structural analysis 19](#_Toc202771162)

[9 Theoretical calculations 21](#_Toc202771163)

[10 References 22](#_Toc202771164)

# Methods

**Nuclear magnetic resonance (NMR) spectra** were recorded on Bruker AV 400 and AV 400 TR spectrometers. Proton chemical shifts are expressed in parts per million (*δ*-scale) and are calibrated using residual non-deuterated solvent peaks as internal reference (^1^H‐NMR: CDCl_3_: 7.26, DMSO‐*d*_6_: 2.50).

**UV-Vis** **spectra** were recorded using a Perkin-Elmer Lambda 1050 spectrometer equipped with a 150 mm integrating sphere. Diffuse reflectance spectra were recorded using a Harrick Praying Mantis accessory kit and were referenced to barium sulfate as the white standard.

High resolution electron ionization (EI) mass spectra (MS) were recorded with a Thermo Finnigan MAT 95 instrument.

**Nitrogen sorption isotherms** were recorded on a Quantachrome Autosorb 1 at 77 K within a pressure range of *p*/*p_0_* = 0.001 to 0.98. Prior to the measurement of the sorption isotherms, the samples were heated for 24 h at 120 °C under turbo-pumped vacuum. For the evaluation of the surface area, the BET model was applied between 0.0005 and 0.08 *p*/*p_0_*. Pore size distributions were calculated using the NLDFT adsorption model for cylindrical pores.

**Powder X-ray diffraction (PXRD)** measurements were performed using a Bruker D8 Discover diffractometer with Ni-filtered Cu K*_α_* radiation and a LynxEye position-sensitive detector.

The initial structure model of the COF was built using the Forcite module of the Accelrys Materials Studio software package. For the COTh-1P COF, we applied a tetragonal crystal system with the highest possible symmetry in the space group *I*4_1_. Experimental PXRD data were used for the respective Rietveld and Pawley refinements to optimize the structure models.

**2D grazing-incidence wide angle X-ray scattering (GIWAXS)** data were recorded with an Anton Paar SAXSpoint 2.0 system equipped with a Primux 100 micro Cu K*_α_* source and a Dectris EIGER R 1M detector. The COF films were positioned at a sample-detector distance of 140 mm and were measured with an incidence angle of 0.2°.

**Transmission electron microscopy** **(TEM)** was performed on a probe-corrected FEI Titan Themis instrument equipped with a field emission gun operated at 300 kV.

**Scanning electron microscopy** **(SEM)** images were recorded with an FEI Helios NanoLab G3 UC scanning electron microscope equipped with a field emission gun operated at 3 – 5 kV.

**Fourier-transform infrared spectroscopy (FT-IR)** measurements were performed with a Bruker Vertex 70 FTIR instrument by focusing light of a globar (silicon carbide) as MIR light source through a KBr beam splitter with integrated gold mirrors and an ATR sample stage with a Ge crystal. The spectra were recorded by a N2 cooled MCT detector with a resolution of 2 cm−1 and averaged over 1000 scans

**Photoluminescence** **(PL)** data were processed with a FluoTime 300 instrument from PicoQuant GmbH. The samples were photo-excited using lasers with suitable wavelengths according to the sample absorption, in this case 378 nm wavelength (LDH-P-C-375 from PicoQuant GmbH) pulsed at 40 MHz, with a pulse duration of ~100 ps and fluence of ~ 300 nJ cm^−2^/pulse. The PL was collected using a high-resolution monochromator and photomultiplier detector assembly (PMA-C 192-N-M, PicoQuant GmbH).

**Cyclic voltammetry** measurements were conducted in a three-electrode-setup with a Metrohm Autolab PGSTAT potentiostat/galvanostat and a set scan speed of 50 mV s^−1^. The COF thin film grown on a conductive ITO substrate was applied as the working electrode and immersed into an electrolyte solution consisting of 0.1 M tetrabutylammonium hexafluorophosphate (TBAPF_6_) in MeCN. A Pt wire and an Ag wire were used as the counter electrode and the pseudoreference electrode, respectively. All potentials are referenced to the fc/fc^+^ redox pair.

**Optical Pump Terahertz Probe (OPTP)** **Spectroscopy.** A full description of the OPTP setup used in this study has been published previously.^[1a]^ Briefly, we use an amplified Ti:sapphire laser system (Spectra-Physics Spitfire) to provide a 35 fs fundamental pulse, with a central wavelength of 800 nm and a 5 kHz repetition rate. This output is used to generate intense THz radiation pulses in a spintronic emitter via the inverse spin Hall effect.^[2]^ We use free-space electro-optic (EO) sampling in a 1 mm-thick ZnTe (110) crystal, a Wollaston prism and a pair of balanced photodiodes to measure the THz transmission. Furthermore, samples are excited by frequency-doubled 400 nm pulses, obtained via second-harmonic generation in a beta-barium-borate (BBO) crystal. Samples for OPTP are blade coated onto 2 mm thick z-cut quartz. During the OPTP measurements, the THz emitter, EO crystal, and samples are kept under vacuum at pressures > 0.1 mbar.

**
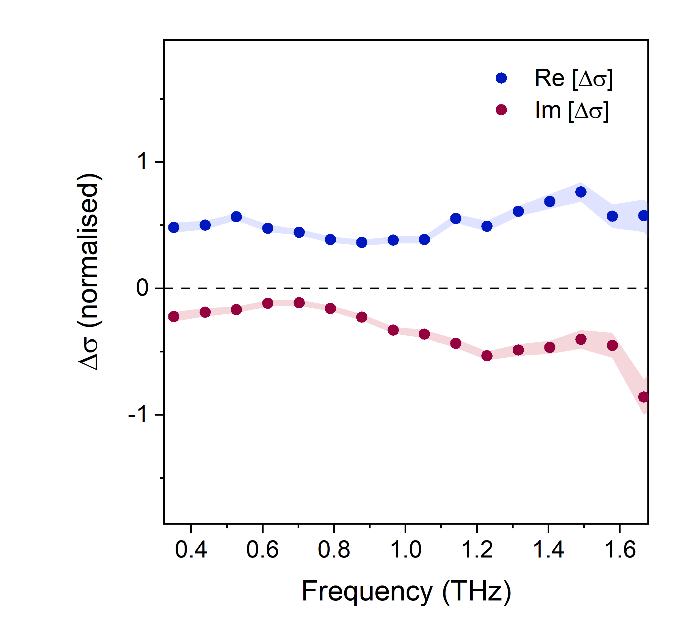
**

**Figure S1**: Complex THz photoconductivity spectrum measured for a COTh-1P-COF thin film at the peak of the photoconductivity signal (t = 0.25 ps) and at a fluence of 60 μJ cm^−2^. Different colors represent the real and imaginary part of the complex THz photoconductivity.

**Derivation of charge-carrier mobility from OPTP measurements.** The effective electron-hole sum mobility was determined using the approach developed by Wehrenfennig et al..^[3]^ Furthermore, we fitted the OPTP transient with a single exponential decay model convoluted with a Gaussian instrumental response function. In the adopted experimental geometry (i.e., transmission geometry for thin film on a z-cut quartz substrate), the measured $\left( \frac{\Delta T}{T} \right)$ signal can be converted to the material sheet photoconductivity $\Delta S$ via the thin-film approximation. The resulting sheet photoconductivity is

| $\Delta S= -\epsilon_{0}c(n_{1}+n_{3})\left( \frac{\Delta T}{T} \right)$ | (S1) |
| --- | --- |

where *n_1_* = 2.13 and *n_3_* = 1 are the refractive indices of quartz and vacuum, respectively.^[4]^

To quantify the charge-carrier mobility, the number of photogenerated charge carriers *N* must be determined. Here, we estimate the areal density of photogenerated carriers, proportional to the number of absorbed photons, as

| $\frac{N}{A_{eff}}= \phi\frac{E\lambda}{hcA_{eff}}\left( 1-R_{pump}-T_{pump} \right)$ | (S2) |
| --- | --- |

Where $\phi$ is the photon-to-charge branching ratio (i.e., the fraction of generated charges per photons

absorbed), *E* is the excitation energy per pulse, and $\varepsilon=hc/\lambda$ is the energy of a photon with wavelength *λ*, and *R_pump_* and *T_pump_* are the reflectance and transmittance at 400 nm (i.e., excitation wavelength). Summarizing this, the effective sum mobility can be then extracted as the ratio between the sheet photoconductivity (Eq. S1) and the areal charge density (S2):

| $\phi\mu= -\epsilon_{0}c(n_{1}+n_{3})\frac{A_{eff}hc}{eE\lambda(1-R_{pump}-T_{pump})}\left( \frac{\Delta T}{T} \right)$ | (S3) |
| --- | --- |

Here, to capture the photoconductivity dynamics in COFs, we fitted the OPTP traces with a single exponential decay function convoluted with a Gaussian instrumental response function ($IRF \approx200 fs$). The OPTP signal is then expressed as

| $\left( \frac{\Delta T}{T} \right)\left( t \right)= A\exp\left( {-k}_{d}t \right) \bigotimes g(t,t_{0},\sigma)$ | (S4) |
| --- | --- |

Where *k_d_* is the decay rate and $g(t,0,\sigma)$ is a Gaussian function centered at *t_0_* with broadening *σ*. The resulting fits are reported in Figure 5.

**Drude Factor.** The Drude model is a simple classical model describing the frequency-dependent conductivity in semiconductors and metals. For photogenerated electrons and holes in an emerging semiconductor, generally exhibiting charge-carrier scattering time in the order of a few femtoseconds,^[5]^ the Drude model predicts zero-valued imaginary and frequency-independent real components of the photoconductivity in the THz range investigated in this work (0.5-2.5 THz).^[6]^ Milot et al.^[1b]^ introduced a *Drude factor* $f_{D}$ to quantify deviations from it, defined as:

| $f_{D}=\frac{1}{n} \sum_{n} \frac{\sqrt{Re\left( {\Delta T}/T \right)^{2}}}{\sqrt{Re\left( {\Delta T}/T \right)^{2}+Im\left( {\Delta T}/T \right)^{2}}}$ | (S5) |
| --- | --- |

Where *n* is the number of frequency points measured in the THz spectrum, and ${\Delta T}/T$ is the measured fractional change in THz transmission at each frequency point.^[1a]^ The Drude factor values can range between 0 and 1, where 1 indicates the ideal Drude conductivity model, while deviations from it can originate from charge-carrier localization and disorder-induced dispersive transport.

**Density functional theory (DFT) calculations** were performed with the Vienna Ab initio Simulation Package (VASP)^[7]^. We used the projector-augmented wave (PAW) method^[8]^ in combination with the Perdew-Burke-Ernzerhof (PBE) exchange correlation functional^[9]^. The atomic positions and the lattice parameters of the molecular structure of the 3D COF were optimized in an alternating fashion with multiple steps, which has been proven to be very effective for COFs.^[10]^ An energy convergence value of 10^-6^ eV and a kinetic energy cutoff of 400 eV (for atom relaxation) and 520 eV (for lattice relaxation) were used. The van der Waals (vdW) dispersion was corrected with the Becke-Johnson damping variant of DFT-D3^[11]^.

The electronic band structure was computed along the high symmetry paths in the Brillouin zone of the primitive tetragonal unit cell. Each segment along the high symmetry paths Γ – X – M – Γ, Z – R – A – Z was sampled by 20 points and the paths Γ – Z, X – R, M – A were sampled by 60 points. To account for the opening of the band gap and estimate the band gap at hybrid-DFT level (Heyd–Scuseria–Ernzerhof (HSE06) functional^[12]^), we employed an empirical constant scissors shift of 0.57 eV^[13]^ determined at the Γ-point. PXRD simulations were performed with the PyXtal package.^[14]^

# Linker synthesis

All reagents were purchased in high-purity grades from commercial suppliers and used as received without further purification, unless stated otherwise. All reactions were conducted in oven-dried glassware under argon atmosphere using standard Schlenk and glove box techniques.

**Figure S2.** Schematic synthesis route for the COTh(CHO)_4_ building block.

**3,3'-Dibromo-[2,2'-bithiophene]-5,5'-dicarbaldehyde (1)**

Into a 250 mL two-necked Schlenk-flask a solution of 3,3',5,5'-tetrabromo-2,2'-bithiophene (5410 mg, 11.2 mmol, 1.0 eq.) in 60 mL anhydrous THF was cooled to −78 °C. *n*-BuLi (2.5 M in hexane, 25.26 mmol, 2.25 eq.) was added dropwise and the solution was stirred for 2 h at −78 °C. Subsequently, anhydrous DMF (2.16 mL, 28.1 mmol, 2.5 eq.) was added and the resulting mixture was allowed to warm to room temperature and stirred overnight. The reaction was then quenched with 80 mL of 6 M HCl and stirred for another hour. The resulting precipitate was collected by filtration, washed with H_2_O and recrystallized in 20 mL DMSO at 180 °C. After cooling to room temperature and washing with MeOH, the product was dried under high vacuum to yield the title compound as yellowish green needles (2270 mg, 5.97 mmol, 53%).

^1^H NMR (400 MHz, DMSO-*d_6_*): 9.97 (s, 2H), 8.26 (s, 2H).

**2,2'-(3,3'-Dibromo-[2,2'-bithiophene]-5,5'-diyl)bis(5,5-dimethyl-1,3-dioxane) (2)**

Into a 100 mL two-necked Schlenk-flask reaction mixture containing compound **1** (1833 mg, 4.82 mmol, 1.0 eq.), 2,2-dimethyl-1,3-propanediol (5023 mg, 48.2 mmol, 10 eq.), and *p*-toluenesulfonic acid monohydrate (73.4 mg, 0.39 mmol, 8 mol%) in 48 mL benzene was refluxed at 85 °C overnight. After cooling to room temperature, water (50 mL) was added and the product was extracted with DCM and washed with brine three times. The combined organic phases were dried over MgSO_4_ and concentrated under reduced pressure. Purification via column chromatography (silica gel, DCM/n-hexane 2:1) yielded the title compound as a pink powder (2267 mg, 4.10 mmol, 85%).

^1^H NMR (400 MHz, CDCl_3_): 7.10 (d, *J* = 0.7 Hz, 2H), 5.58 (d, *J* = 0.8 Hz, 2H), 3.76 (d, *J* = 11.3 Hz, 4H), 3.63 (d, *J* = 10.7 Hz, 4H), 1.26 (s, 6H), 0.80 (s, 6H).

^13^C NMR (101 MHz, CDCl_3_): 143.15, 129.21, 128.67, 111.93, 97.55, 77.62, 30.40, 23.10, 21.95.

**2,5,8,11-Tetrakis(5,5-dimethyl-1,3-dioxan-2-yl)cyclooctatetrathiophene (3) ^[15]^**

Into a 250 mL three-necked Schlenk-flask compound **2** (2267 mg, 4.10 mmol, 1.0 eq.) was dissolved in 40 mL THF and 40 mL diethyl ether. After cooling to -78 °C, *n*-BuLi (2.5 M, 9.44 mmol, 2.3 eq.) was added dropwise and the resulting mixture was stirred for 2 hours. Following the addition of anhydrous CuCl_2_ (1655 mg, 12.31 mmol, 3.0 eq.), the reaction was allowed to warm to room temperature and stirred overnight. The reaction was quenched with H_2_O and stirred for 2 h. The product was extracted with DCM and washed with brine (3x). The organic phase was dried over MgSO_4_ and concentrated under reduced pressure. Purification via column chromatography (silica gel, DCM + 1% EtOAc) yielded the product as a yellow powder (1211 mg, 1.54 mmol, 75%).

^1^H NMR (400 MHz, CDCl_3_): 6.96 (s, 4H), 5.55 (s, 4H), 3.77 – 3.68 (m, 8H), 3.64– 3.53 (m, 8H), 1.25 (s, 12H), 0.78 (s, 12H).

^13^C NMR (101 MHz, CDCl_3_): 142.64, 136.33, 132.78, 127.64, 98.03, 77.53, 30.34, 23.09, 21.99.

**Cycloocta[1,2-b:4,3-b':5,6-b'':8,7-b''‘] tetrathiophene-2,5,8,11-tetracarbaldehyde (COTh(CHO)_4_)**

Into a 250 mL round bottom flask compound **3** (819 mg, 1.04 mmol, 1.0 eq.) was dissolved in 75 mL CHCl_3_. After adding 1.5 mL H_2_O and 15 mL trifluoroacetic acid, the reaction mixture was stirred under argon for 2 h. The reaction mixture was then slowly added to 500 mL saturated aqueous NaHCO_3_ solution and extracted with DCM. The organic phase was thoroughly washed with H_2_O, dried over MgSO_4_ and concentrated under reduced pressure. Purification by column chromatography (silica gel, DCM + 7% EtOAc) yielded the title compound as a bright yellow powder (225 mg, 0.51 mmol, 49%).

^1^H NMR (400 MHz, DMSO): 9.96 (s, 4H), 8.14 (s, 4H).

^13^C NMR (101 MHz, DMSO): 184.33, 145.16, 139.43, 138.66, 136.63.

HRMS(EI) calcd for C_20_H_8_O_4_S_4_ : 439.9305: found 440.9297

**Figure S3.** ^1^H Spectrum of COTh(CHO)_4_.

**Figure S4.** ^13^C Spectrum of COTh(CHO)_4_.


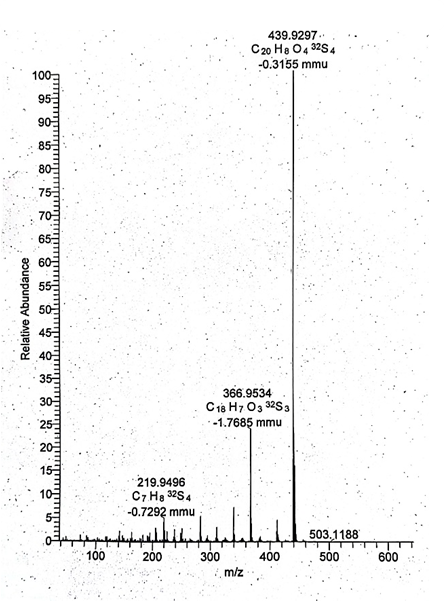


**Figure S5**. HRMS(EI) of COTh(CHO)_4_ building block.

# COF Synthesis

The preparation and reaction of the COF syntheses was conducted under argon atmosphere. Solvents and acetic acid were obtained in high purity grade from commercial suppliers and were, unless shipped under inert gas, degassed and flushed with argon prior to use.

## COTh-1P COF powder

COTh(CHO)_4_ (8.82 mg, 20 µmol, 1.0 eq.), aniline (36.5 µL, 400 µmol, 20 eq.), benzyl alcohol (500 µL) and 6 M acetic acid (100 µL) were filled into a 5 mL culture tube under argon conditions. Subsequently, *p*-phenylenediamine (4.32 mg, 40 µmol, 2.0 eq.) and additional 500 µL benzyl alcohol were added. The reaction tube was sealed and heated at 120 °C for 10 d. After cooling to room temperature, the precipitate was filtrated and washed with dry tetrahydrofuran. Extraction with supercritical CO_2_ yielded the COTh-1P COF as a dark-red powder.

## COTh-1P film

COTh(CHO)_4_ (2.21 mg, 5 µmol, 1.0 eq.), aniline (9.13 µL, 100 µmol, 20 eq.), benzyl alcohol (1000 µL) and 6 M acetic acid (200 µL) were filled into a 100 mL screw-cap glass autoclave. Following the addition of *p*-phenylenediamine (1.08 mg, 10 µmol, 2.0 eq.), the reaction mixture was dispersed in additional 1000 µL benzyl alcohol. Subsequently, fused silica or ITO/glass substrates (1.5 x 1 cm) were inserted horizontally in PTFE holders with the activated (plasma-cleaned) side face-down. Prior to use, the substrates were cleaned in a Hellmanex III detergent solution (0.5% *v/v*), deionised water, acetone, and isopropanol, and then activated with an O_2_-plasma. The autoclave was sealed and heated at 120 °C for 5 d. After cooling to room temperature, the substrate with the COF film was removed from the reaction solution, immersed in anhydrous THF and dried with a nitrogen stream

## Effect of modulator


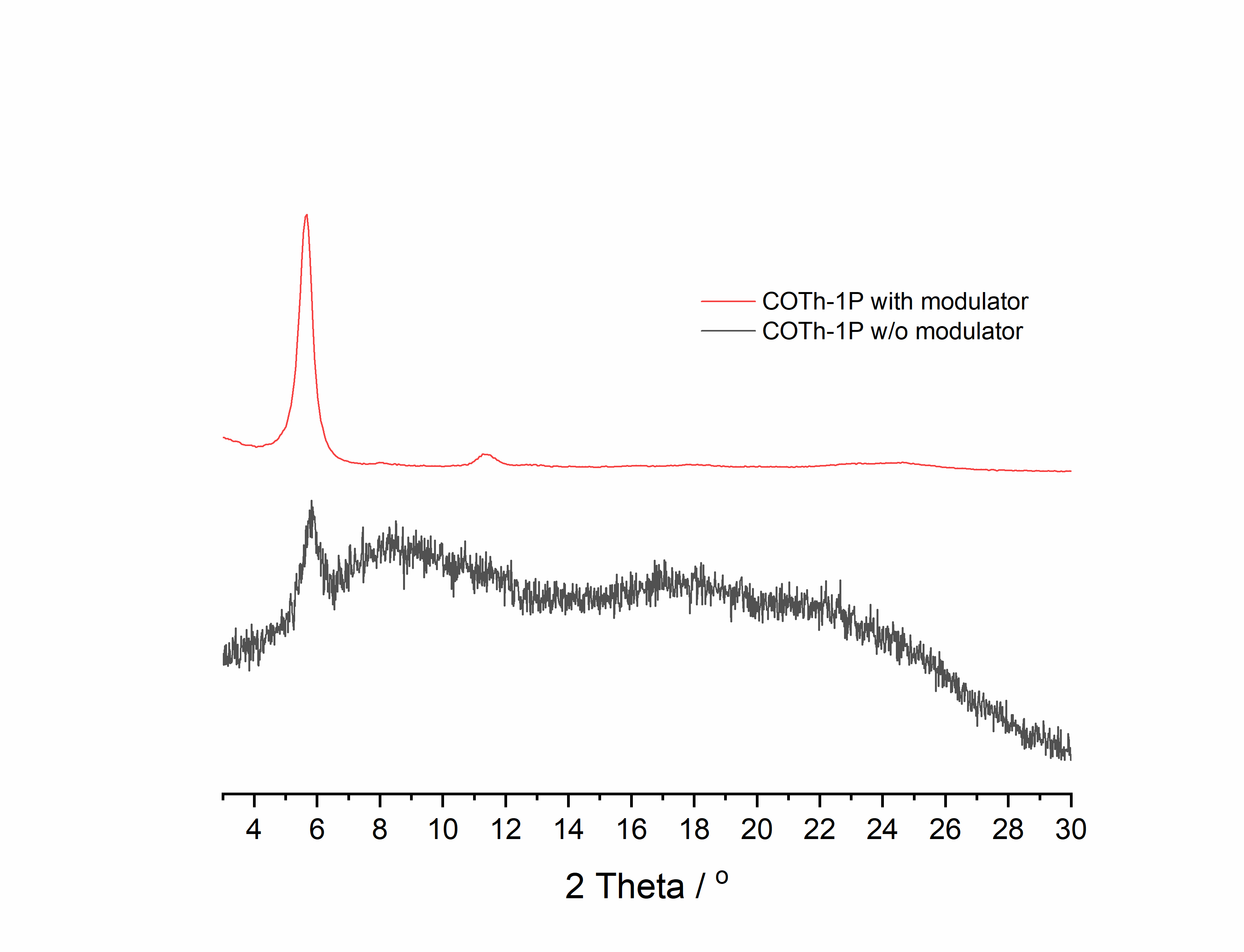


**Figure S6.** Comparison of the PXRD patterns of COTh-1P COF synthesized using a modulator (red line) and without a modulator (black line). The sample prepared with a mono-functionalized modulator exhibits significantly enhanced crystallinity, as evidenced by well-defined, high-intensity reflections at low angles and a markedly reduced background signal.

| **Modulator: Aniline** | | | | | |
| --- | --- | --- | --- | --- | --- |
| **Entry** | **Modulator eq.** | **Modulator volume**  **(µL)** | **COTh(CHO)_4_**  **(µmol)** | **PPD**  **(µmol)** | **Volume BnOH**  **(µL)** |
| 1A | 5 | 2.2 | 5 | 10 | 250 |
| 1B | 10 | 4.5 | 5 | 10 | 250 |
| 1C | 15 | 6.8 | 5 | 10 | 250 |
| **1D**  **(Conditions reported)** | **20** | **9.1** | **5** | **10** | **250** |
| 1E | 25 | 12.4 | 5 | 10 | 250 |

PPD: *p*-phenylene diamine

BnOH: Benzyl alcohol

**Table S1.** Screening conditions using aniline as modulator.





**Figure S7.** PXRD patterns of COTh-1P COF synthesized using different equivalents of aniline as a modulator, as shown above in Table S1. The data show that the reported synthesis conditions are optimized with aniline serving as modulator.

| **Modulator: *p*-Anisidine** | | | | | |
| --- | --- | --- | --- | --- | --- |
| **Entry** | **Modulator eq.** | **Modulator mass**  **(mg)** | **COTh(CHO)_4_**  **(µmol)** | **PPD**  **(µmol)** | **Volume BnOH**  **(µL)** |
| 2A | 5 | 3 | 5 | 10 | 250 |
| 2B | 10 | 6 | 5 | 10 | 250 |
| 2C | 15 | 9 | 5 | 10 | 250 |
| 2D | 20 | 12 | 5 | 10 | 250 |

PPD: *p*-phenylene diamine

BnOH: Benzyl alcohol

**Table S2.** Screening conditions using *p-anisidine* as modulator.

**

**

**Figure S8.** PXRD patterns of COTh-1P COF synthesized using different equivalents of *p*-anisidine as a modulator, compared to the above optimized conditions with aniline serving as modulator. The data show that p-anisidine does not improve crystallinity compared to aniline.

| **Entry** | **Modulator** | **Modulator eq.** | **COTh(CHO)_4_**  **(µmol)** | **PPD**  **(µmol)** | **Volume BnOH**  **(mL)** |
| --- | --- | --- | --- | --- | --- |
| **A** | - | 0 | 5 | 10 | 2 |
| **B** | Aniline | 30 | 5 | 10 | 2 |
| **C** | *p*-Anisidine | 20 | 5 | 10 | 2 |
| **D** | Benzaldehyde | 20 | 5 | 10 | 2 |

PPD: *p*-phenylene diamine

BnOH: Benzyl alcohol

**Table S3.** Screening conditions of film synthesis using different modulators.


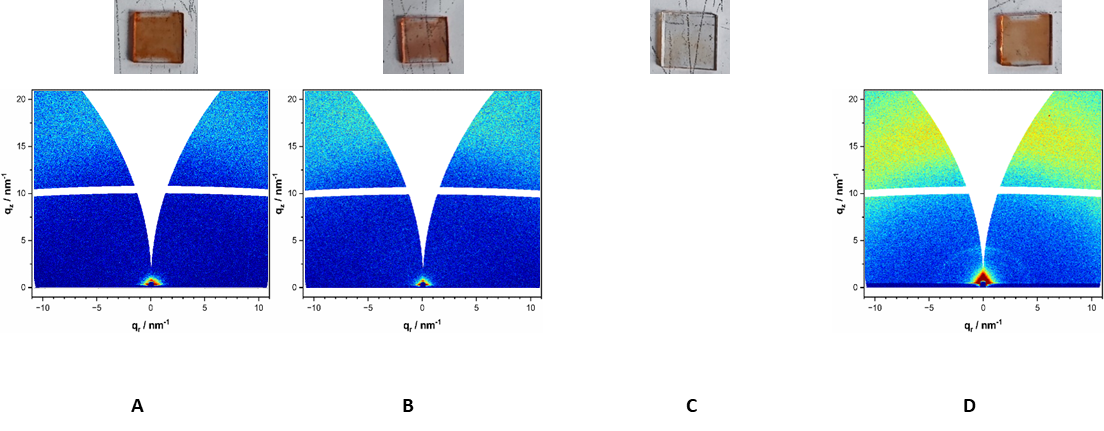


**Figure S9.** Additional conditions used for screening the synthesis of COTh-1P films (top) and GIWAXS patterns obtained (bottom) for the different films. Conditions A-D correspond to entries A-D in Table S3. For condition C, GIWAXS measurements were not performed due to the absence of film formation. As shown in the images and the corresponding GIWAXS patterns, none of the alternative conditions tested led to an improvement over those presented in the main text. These findings support the conclusion that the use of 20 equivalents of aniline as modulator is critical for obtaining a highly crystalline and oriented thin film of the COTh-1P COF.

# Optical Characterization


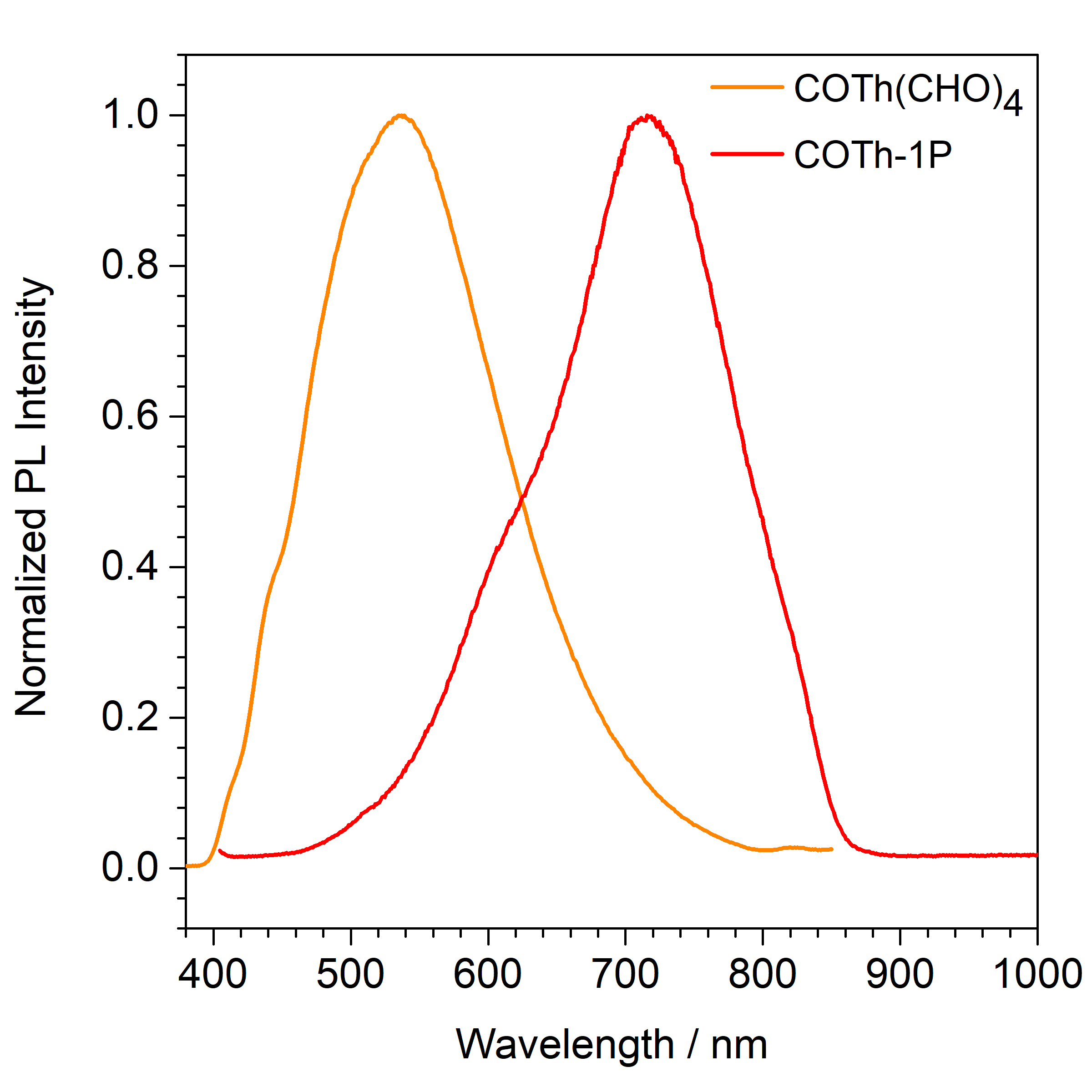


**Figure S10.** Photoluminescence spectra of the COTh(CHO)_4_ building block measured in 50 *µ*M CHCl_3_ solution and the COTh-1P COF thin film.


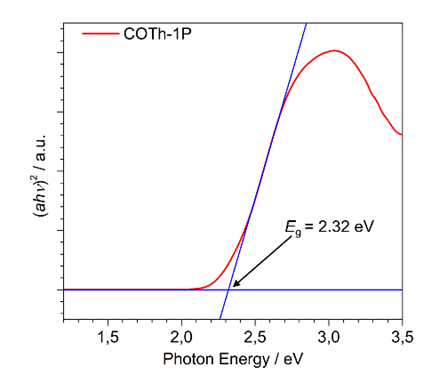


**Figure S11.** Band gap determination via Tauc plot for COTh-1P COF, assuming a direct band gap.

# Electrochemical Measurements


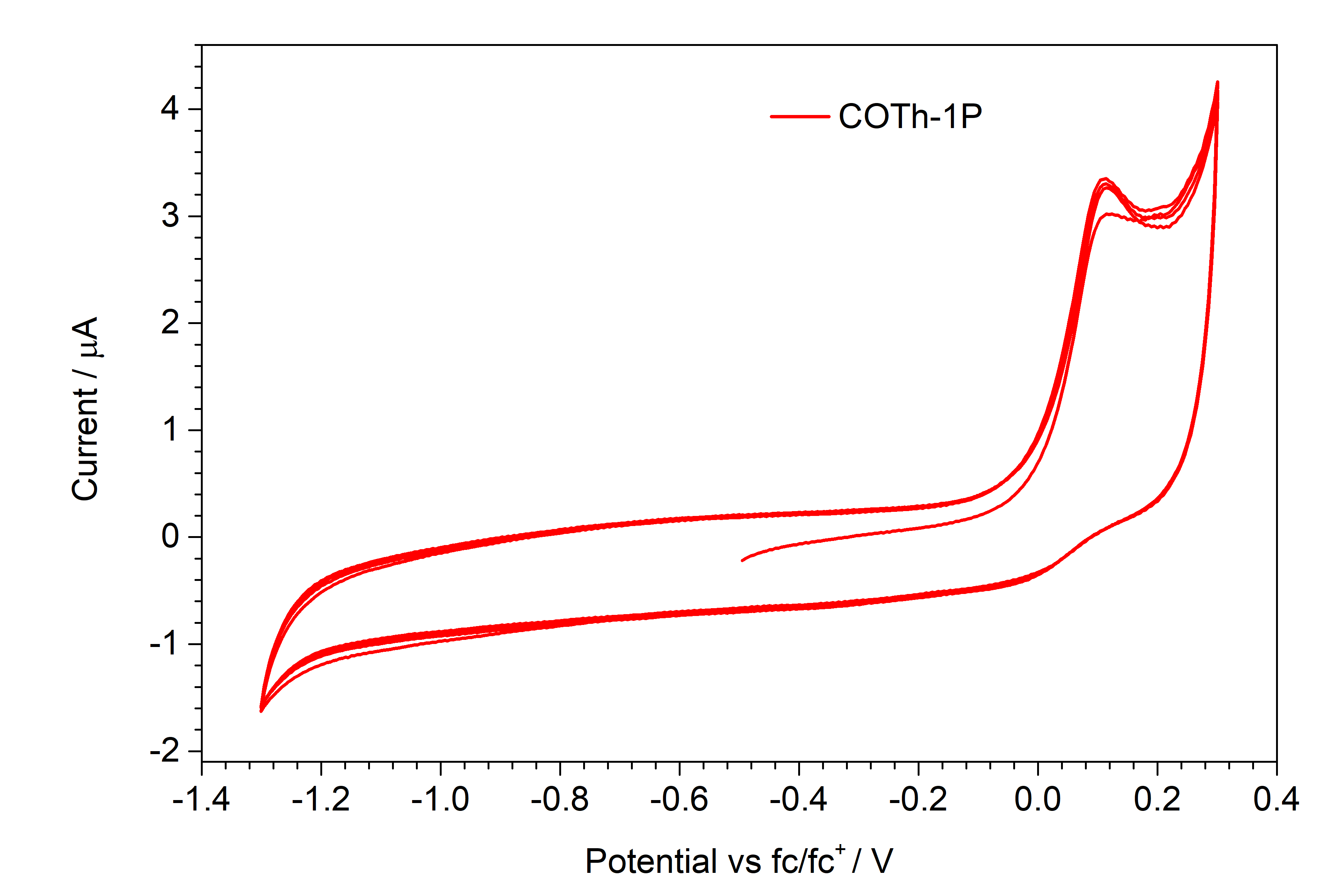


**Figure S12.** Cyclic voltammetry scans of the COTh-1P COF show a distinct oxidation wave at 0.11 V. Minimal drift over four cycles shows the electrochemical stability of the COF within this potential range.

# Scanning electron microscopy


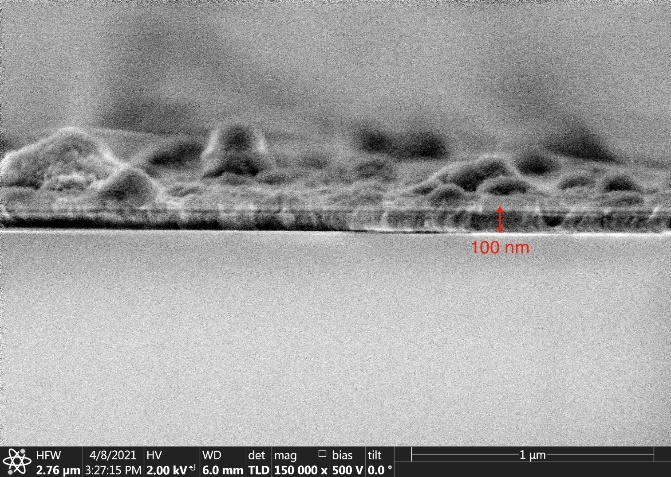

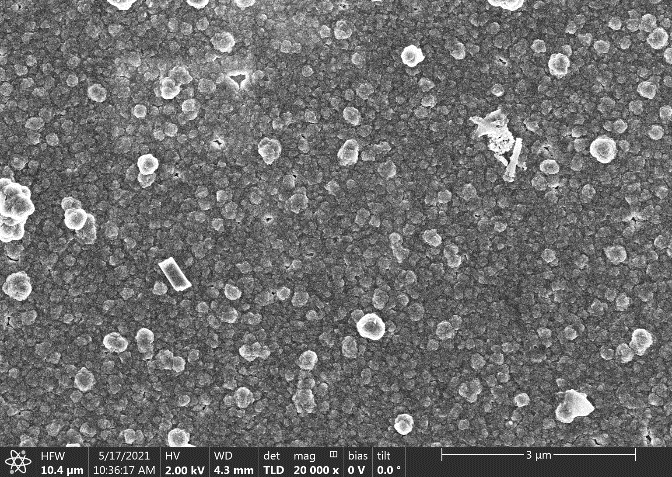


**Figure S13.** Cross-section (left) SEM images of the COTh-1P COF thin film with 100 nm thickness,
and top-view (right).

# Fourier-transform infrared spectroscopy (FT-IR)

**
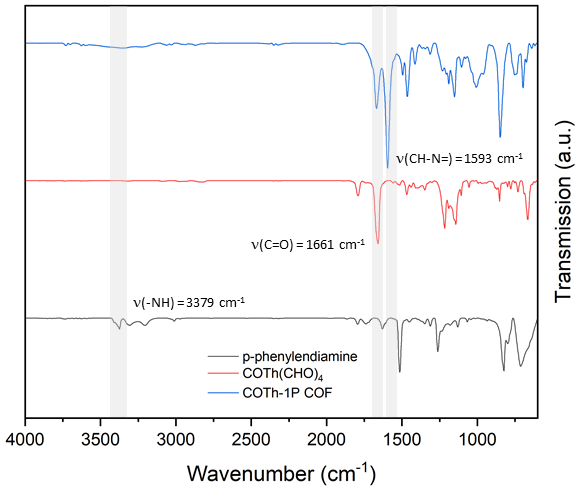
**

**Figure S14**. FT-IR spectra of building blocks and COTh-1P COF, respectively.

# Structural analysis

**
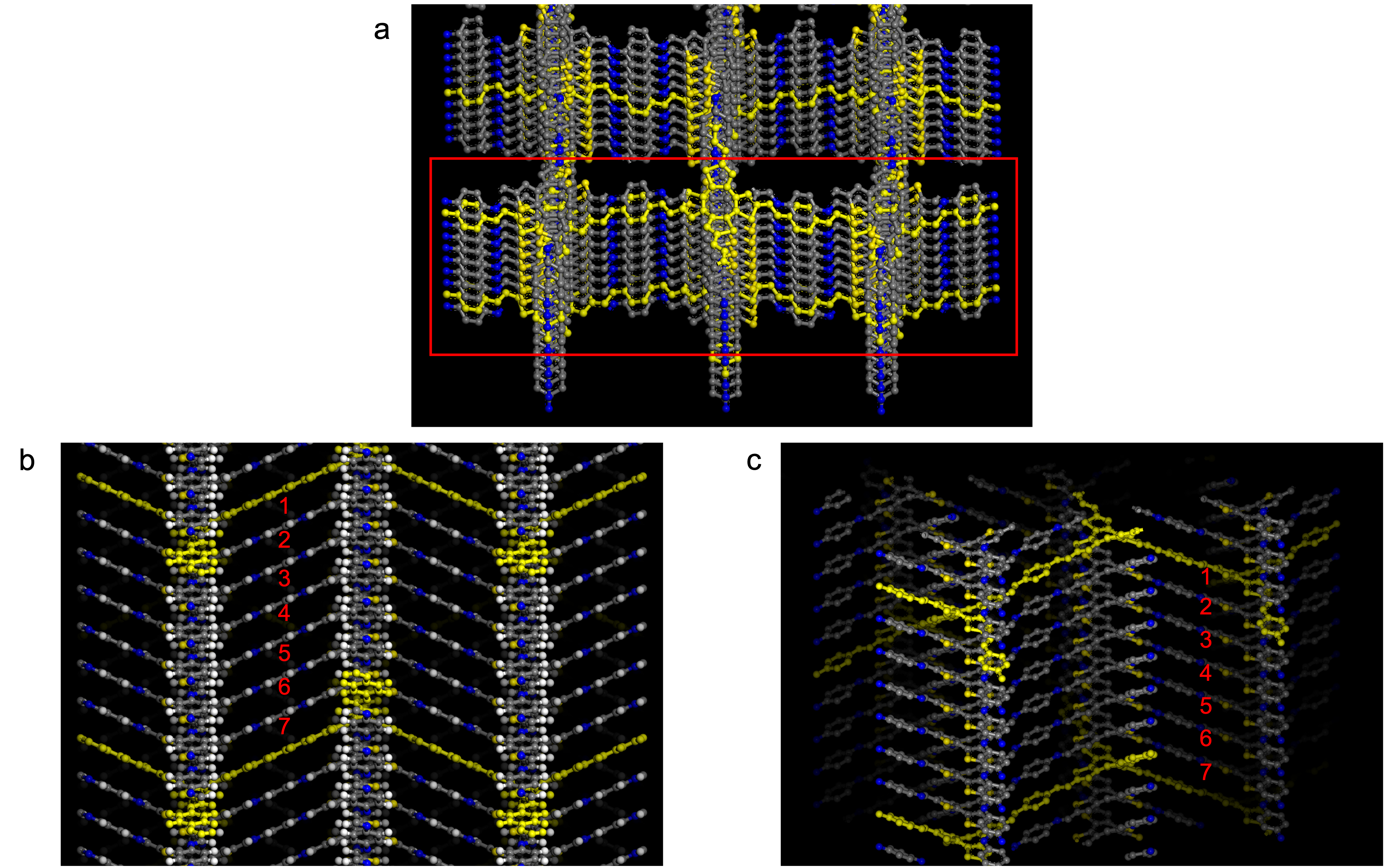
**

**Figure S15.** Visualization of the interpenetration level of the COTh-1P COF. (**a**) Tilted side view onto the structure simulation with a single fragment highlighted (yellow). The red box marks the segment that is magnified for determining the degree of interpenetration. The magnified view onto the structure model along the *b*-axis (**b**) and the tilted view onto the framework edge (**c**) clearly shows the 7-fold interpenetration of the 3D COF.

| **Unit cell parameters (I4_1_) and atomic coordinates for COTh-1P COF** | | | |
| --- | --- | --- | --- |
| **a = b = 30.96 Å, c = 4.59 Å** | | | |
|  |  | **α = β = γ = 90.0000^o^** |  |
| **Atom** | **a/x** | **b/y** | **c/z** |
| C1 | 0.55445 | 0.51418 | 0.57797 |
| S2 | 0.60138 | 0.52934 | 0.73842 |
| C3 | 0.60917 | 0.47614 | 0.82985 |
| C4 | 0.57853 | 0.44747 | 0.73040 |
| C5 | 0.54686 | 0.46939 | 0.58537 |
| N6 | 0.67473 | 0.49268 | 0.06137 |
| C7 | 0.64691 | 0.46281 | -0.00831 |
| C8 | 0.73933 | 0.52320 | 0.26574 |
| C9 | 0.72511 | 0.44591 | 0.32844 |
| C10 | 0.71320 | 0.48659 | 0.21891 |
| C11 | 0.44562 | 0.98557 | 0.67339 |
| C12 | 0.40874 | 0.97419 | 0.52961 |
| C13 | 0.38960 | 0.01068 | 0.41824 |
| S14 | 0.41430 | 0.05882 | 0.49462 |
| C15 | 0.45321 | 0.03038 | 0.66808 |
| N16 | 0.32768 | 0.97602 | 0.19643 |
| C17 | 0.35020 | 0.01077 | 0.25316 |
| C18 | 0.26313 | 0.94240 | 0.98501 |
| C19 | 0.27693 | 0.01973 | 0.92069 |
| C20 | 0.28923 | 0.97937 | 0.03259 |
| H21 | 0.58162 | 0.41091 | 0.7728 |
| H22 | 0.65216 | 0.42739 | 0.05623 |
| H23 | 0.72989 | 0.55625 | 0.17668 |
| H24 | 0.70391 | 0.41582 | 0.29012 |
| H25 | 0.39823 | 0.93843 | 0.51682 |
| H26 | 0.33750 | 0.04281 | 0.16535 |
| H27 | 0.27275 | 0.90936 | 1.07356 |
| H28 | 0.29789 | 0.05006 | 0.95667 |

# Theoretical calculations

For the DFT calculations, the lowest-energy structure was obtained, exhibiting lattice parameters of *a* = *b* = 31.8 Å, *c* = 4.42 Å, and α = β = γ = 90°. These parameters are in good agreement with those reported in the manuscript. The simulated PXRD pattern of this relaxed structure shows reasonable agreement with the experimental data, particularly in the 23–25° region, which is characteristic of π-stacking of the COTh units along the *c*-direction (Figure S16).


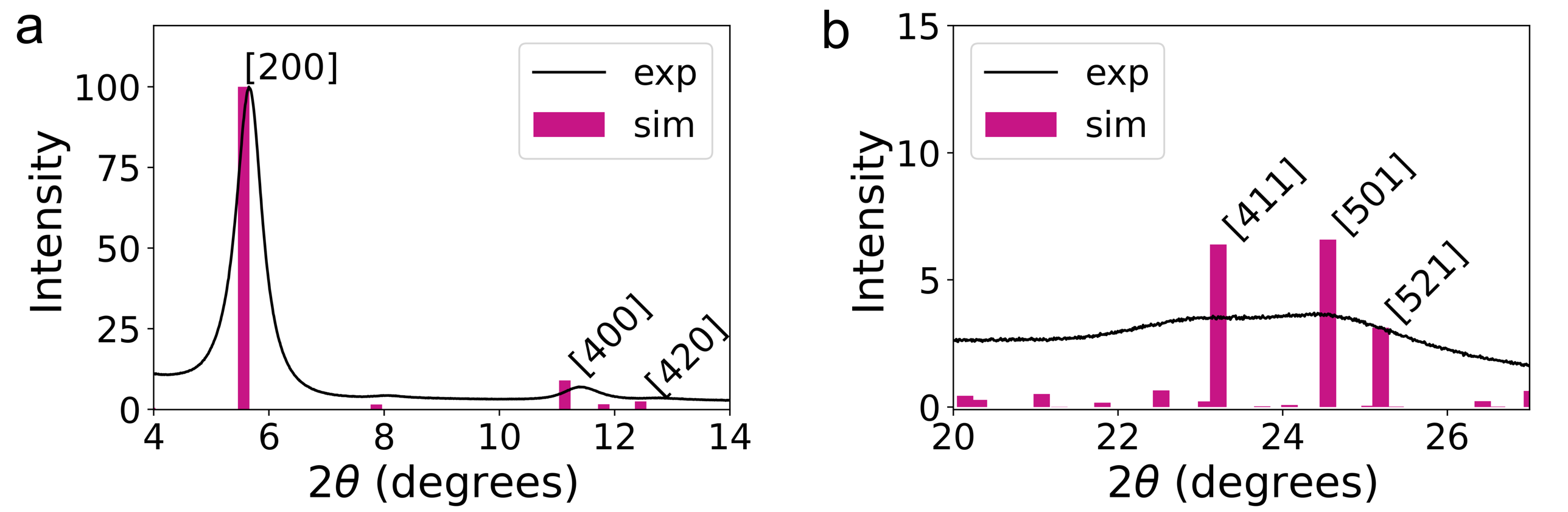


**Figure S16.** Comparison of simulated lowest-energy structure and experimental PXRD data. a) *2θ* Region between 4^o^ and 14^o^. b) *2θ* Region between 20^o^ and 27^o^.

# References

[1] (a) L. R. V. Buizza, A. D. Wright, G. Longo, H. C. Sansom, C. Q. Xia, M. J. Rosseinsky, M. B. Johnston, H. J. Snaith, L. M. Herz, *ACS Energy Letters* **2021**, *6*, 1729-1739. (b) R. L. Milot, G. E. Eperon, H. J. Snaith, M. B. Johnston, L. M. Herz, *Advanced Functional Materials* **2015**, *25*, 6218-6227.

[2] T. Seifert, S. Jaiswal, U. Martens, J. Hannegan, L. Braun, P. Maldonado, F. Freimuth, A. Kronenberg, J. Henrizi, I. Radu, E. Beaurepaire, Y. Mokrousov, P. M. Oppeneer, M. Jourdan, G. Jakob, D. Turchinovich, L. M. Hayden, M. Wolf, M. Münzenberg, M. Kläui, T. Kampfrath, *Nature Photonics* **2016**, *10*, 483-488; F. M. Wagner, S. Melnikas, J. Cramer, D. A. Damry, C. Q. Xia, K. Peng, G. Jakob, M. Kläui, S. Kičas, M. B. Johnston, *Journal of Infrared, Millimeter, and Terahertz Waves* **2023**, *44*, 52-65.

[3] C. Wehrenfennig, G. E. Eperon, M. B. Johnston, H. J. Snaith, L. M. Herz, *Advanced Materials* **2014**, *26*, 1584-1589.

[4] H. J. Joyce, J. L. Boland, C. L. Davies, S. A. Baig, M. B. J. S. S. Johnston, Technology, **2016**, *31*, 103003.

[5] H. Hempel, T. J. Savenjie, M. Stolterfoht, J. Neu, M. Failla, V. C. Paingad, P. Kužel, E. J. Heilweil, J. A. Spies, M. Schleuning, J. Zhao, D. Friedrich, K. Schwarzburg, L. D. A. Siebbeles, P. Dörflinger, V. Dyakonov, R. Katoh, M. J. Hong, J. G. Labram, M. Monti, E. Butler-Caddle, J. Lloyd-Hughes, M. M. Taheri, J. B. Baxter, T. J. Magnanelli, S. Luo, J. M. Cardon, S. Ardo, T. Unold, **2022**, *12*, 2102776; H. Hempel, T. J. Savenjie, M. Stolterfoht, J. Neu, M. Failla, V. C. Paingad, P. Kužel, E. J. Heilweil, J. A. Spies, M. Schleuning, J. Zhao, D. Friedrich, K. Schwarzburg, L. D. A. Siebbeles, P. Dörflinger, V. Dyakonov, R. Katoh, M. J. Hong, J. G. Labram, M. Monti, E. Butler-Caddle, J. Lloyd-Hughes, M. M. Taheri, J. B. Baxter, T. J. Magnanelli, S. Luo, J. M. Cardon, S. Ardo, T. Unold, *Advanced Energy Materials* **2022**, *12*, 2102776.

[6] A. M. Ulatowski, L. M. Herz, M. B. Johnston, *Journal of Infrared, Millimeter, and Terahertz Waves* **2020**, *41*, 1431-1449; R. Ulbricht, E. Hendry, J. Shan, T. F. Heinz, M. Bonn, *Reviews of Modern Physics* **2011**, *83*, 543-586.

[7] G. Kresse, J. Furthmüller, *Computational Materials Science* **1996**, *6*, 15-50; G. Kresse, J. Furthmüller, *Physical Review B* **1996**, *54*, 11169-11186; G. Kresse, J. Hafner, *Physical Review B* **1993**, *47*, 558-561.

[8] P. E. Blöchl, *Physical Review B* **1994**, *50*, 17953-17979; G. Kresse, D. Joubert, *Physical Review B* **1999**, *59*, 1758-1775.

[9] J. P. Perdew, K. Burke, M. Ernzerhof, *Physical Review Letters* **1996**, *77*, 3865-3868.

[10] L. Spies, A. Biewald, L. Fuchs, K. Merkel, M. Righetto, Z. Xu, R. Guntermann, R. Hooijer, L. M. Herz, F. Ortmann, J. Schneider, T. Bein, A. Hartschuh, *Journal of the American Chemical Society* **2025**, *147*, 1758-1766; B. B. Rath, L. Fuchs, F. Stemmler, A. Rodríguez-Camargo, Y. Wang, M. F. X. Dorfner, J. Olbrich, J. van Slageren, F. Ortmann, B. V. Lotsch, *Journal of the American Chemical Society* **2025**.

[11] S. Grimme, S. Ehrlich, L. Goerigk, **2011**, *32*, 1456-1465.

[12] A. V. Krukau, O. A. Vydrov, A. F. Izmaylov, G. E. Scuseria, *The Journal of Chemical Physics* **2006**, *125*.

[13] F. Bechstedt, *Many-Body Approach to Electronic Excitations, Vol. 181*, **2015**.

[14] S. Fredericks, K. Parrish, D. Sayre, Q. Zhu, *Computer Physics Communications* **2021**, *261*, 107810.

[15] J. Urieta-Mora, I. García-Benito, I. Zimmermann, J. Aragó, J. Calbo, G. Grancini, A. Molina-Ontoria, E. Ortí, N. Martín, M. K. Nazeeruddin, *Journal of Materials Chemistry C* **2019**, *7*, 6656-6663.
